# Feature-tracking myocardial strain in healthy adults- a magnetic resonance study at 3.0 tesla

**DOI:** 10.1038/s41598-019-39807-w

**Published:** 2019-03-01

**Authors:** Kenneth Mangion, Nicole M. M. Burke, Christie McComb, David Carrick, Rosemary Woodward, Colin Berry

**Affiliations:** 1 British Heart Foundation Glasgow Cardiovascular Research Centre, University of Glasgow, Glasgow, UK; 2 West of Scotland Heart and Lung Centre, Golden Jubilee National Hospital, Clydebank, UK; 3 Clinical Physics, NHS Greater Glasgow and Clyde, Glasgow, UK

## Abstract

We analyzed feature-tracking derived circumferential and longitudinal strain in healthy volunteers who underwent cardiac magnetic resonance imaging (CMR) at 3.0 T. 88 healthy adults (44.6 ± 18.0 years old, 49% male), without prior cardiovascular disease, underwent CMR at 3.0 T including cine, and late gadolinium enhancement in subjects >45 years. LV functional analysis and feature-tracking strain analyses were carried out. Global strain had better reproducibility than segmental strain. There was a sex specific difference global longitudinal strain (mean ± SD, −18.48 ± 3.65% (male), −21.91 ± 3.01% (female), p < 0.001), but not global circumferential strain (mean ± SD, −25.41 ± 4.50% (male), −27.94 ± 3.48% (female), p = 0.643). There was no association of strain with ageing after accounting for sex for both global longitudinal and circumferential strain. Feature-tracking strain analysis is feasible at 3.0 T. Healthy female volunteers demonstrated higher magnitudes of global longitudinal strain when compared to male counterparts. Whilst global cine-strain has good reproducibility, segmental strain does not.

## Introduction

One of the most important components of a clinical imaging study is the assessment of left ventricular (LV) pump function. The LV ejection fraction (LVEF) is the difference between LV end-diastolic and systolic volumes, divided by the LV end-diastolic volume, hence myocardial contractibility is not directly assessed. Whilst echocardiography is the standard of care, cardiovascular magnetic resonance (CMR) is regarded as the gold-standard for LV functional assessment^1^. The LVEF can be within normal reference ranges in a number of pathological states, which might otherwise have abnormal peak systolic strain values, i.e. identify sub-clinical LV dysfunction. The LVEF cannot be used to provide a detailed assessment of cardiac mechanics due to the complex architectural arrangement of myofibers in circumferential and longitudinal directions. Strain is described as local shortening, thickening and lengthening of the myocardium as a measure of regional and global LV function^2^. Circumferential and longitudinal strain are denoted as negative magnitudes of strain to reflect shortening, whilst radial strain is positive as it reflects myocardial thickening^3^.

Feature-tracking is a technique which has gained traction since being described by Hor *et al*., in 2011^4^, and has resulted in the assessment of myocardial strain from routinely acquired cine imaging sequences in a myriad of pathologies^5–10^. The estimation of strain is reasonably quick^11^. Feature-tracking algorithms are designed to focus on border displacement, with a stronger weighing of endocardial deformation explaining some of the differences in results found in direct comparisons of feature-tracking and other strain modalities^12,13^. Feature-tracking uses optical flow^14^ to track myocardial borders, by tracking a number of points using both 1D and 2D techniques through the cardiac cycle^4^. Feature-tracking has been clinically validated against tagging^13–16^.

There has been an increase in clinical cardiac MR imaging performed at 3.0T^17^. There are a number of advantages to utilizing 3.0 T MR scanners, notably an increase in signal-to-noise ratio, and image resolution^18,19^. Schuster *et al*.^20^ reported that intra-observer variability in cine-strain assessment with feature-tracking at 3.0 T is similar to that observer at 1.5 T. However, balanced steady state free precession imaging is more likely to experience artifact related to the high magnetic field^21^ and feature-tracking reference ranges for healthy volunteers at 3.0 T are currently unavailable with clinically approved (for example, Food and Drug Administration approved) feature-tracking software. A recent meta-analysis^22^ on cine-strain has reported there has been one publication looking at strain using an investigational tissue-tracking software in healthy Chinese volunteers^23^.

There is an increasing body of evidence of the incremental utility of strain in patients with dilated cardiomyopathy^24^, post myocardial infarction^10,25^, and congenital heart disease^5^. Health volunteer reference ranges are required to identify minor reductions in strain parameters when conventional parameters of function, such as the LV ejection fraction are unchanged^15,26,27^.

We aimed to assess circumferential and longitudinal myocardial strain utilizing feature-tracking at 3.0 T in healthy volunteers to provide reference ranges and to investigate the influence of age and sex on strain. We did not investigate radial strain due to reported inferior reproducibility^15,28,29^.

## Results

### Characteristics of The Study Participants

The characteristics of the participants (n = 88) and their LV mass and function are presented in Table 1.

**Table 1:** Characteristics of the healthy volunteers (n = 88).

Characteristic	
Age (years)*	44.6 ± 18.0
Sex (Male) n (%)	43 (49)
Height (cm)*	170 ± 10
Weight (kg)*	75.8 ± 15.0
Body mass index, kgm^−2^	26 ± 4
Body surface area (m^2^)*	1.87 ± 0.21
Imaging parameters	
LVEF (%)	63.6 ± 5.2
LVEDV index (mL/m^2^)	70.1 ± 11.3
LVESV index (mL/m^2^)	25.8 ± 6.6
LV mass index (g/m^2^)	40.4 ± 9.7

*Mean ± SD. LVEF: Left ventricle ejection fraction; LVESV: Left ventricle end-diastolic volume; LVESV: Left ventricle end-systolic volume.

### Inter-observer Analysis

**Figure 1:**
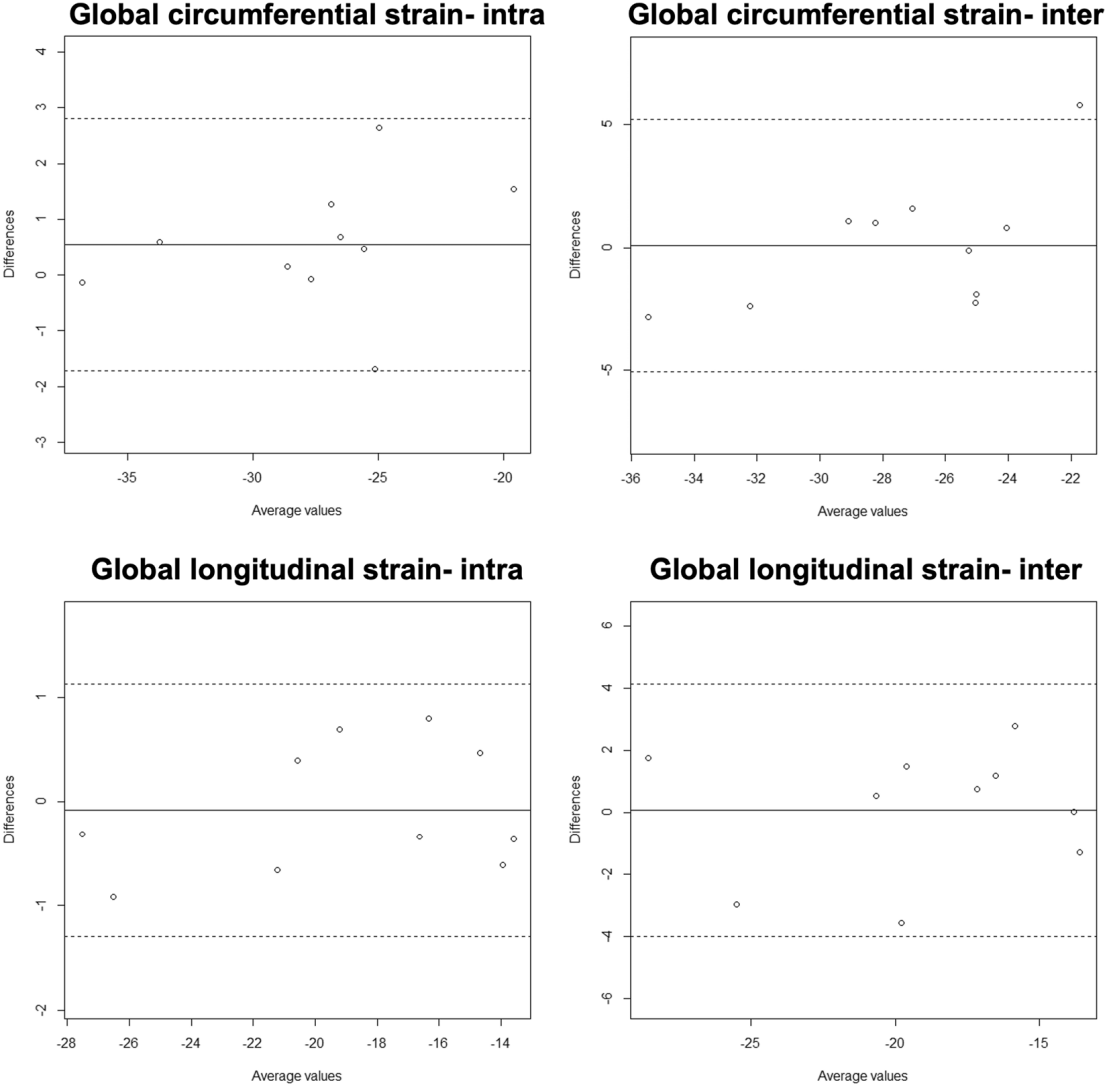
Bland-Altman plots for global strain.

**Figure 2:**
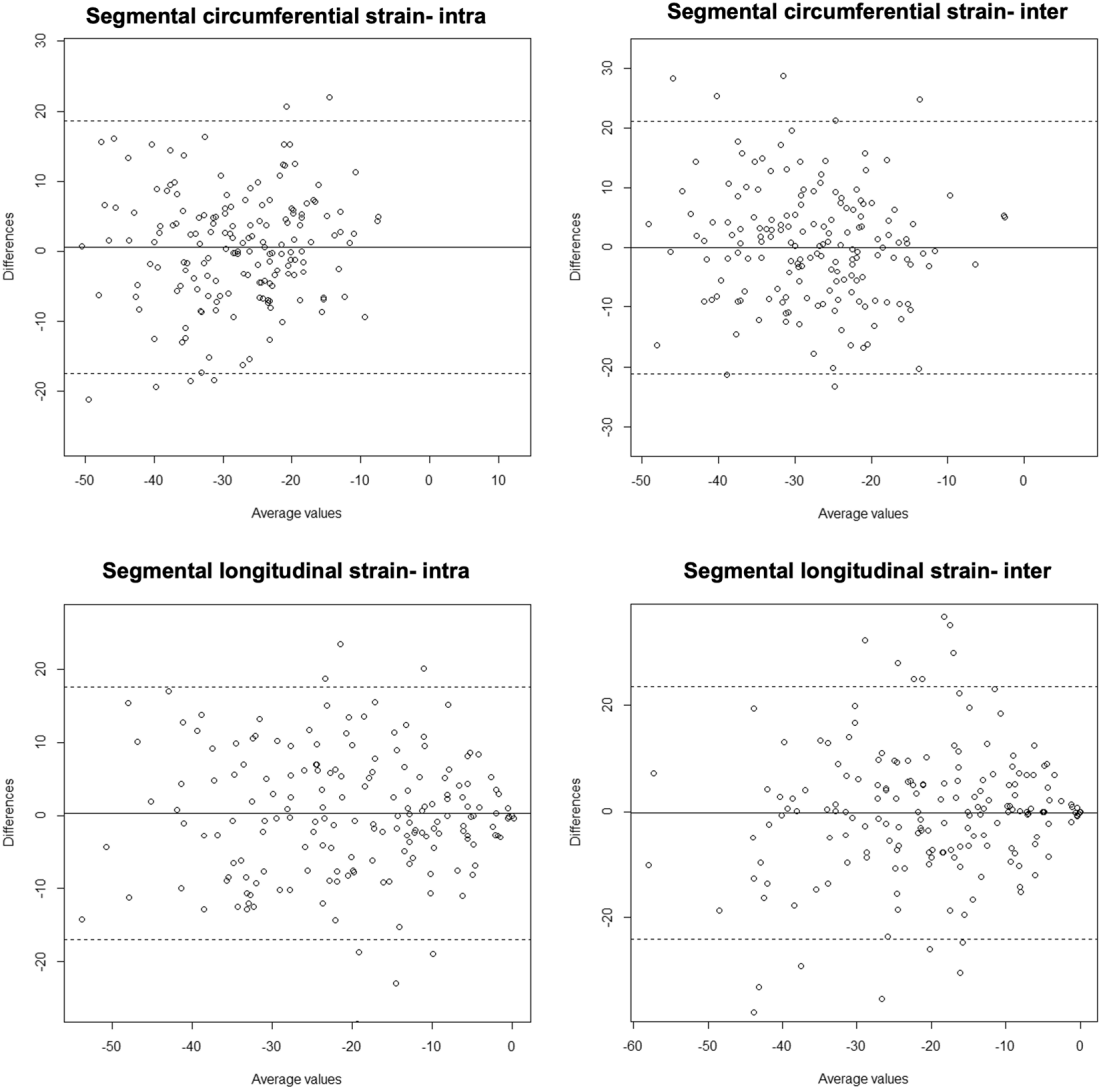
Bland-Altman plots for segmental strain.

All cine imaging was of diagnostic quality. Global longitudinal strain had excellent reproducibility both with inter- and intra-observer analyses and strong positive correlations. Global circumferential strain had excellent reproducibility, as identified by the intra-class correlation co-efficient and strong positive correlations (Fig. 1). Segmental longitudinal strain analyses had a higher bias than global strain analyses, with lower intra-class correlation co-efficient for both intra- and inter-observer analyses. Correlations between reproducibility analyses for segmental longitudinal strain were moderately strong (Table 2). Segmental circumferential strain reproducibility analyses revealed higher biases than global circumferential strain parameters, with a lower intra-class correlation co-efficient, and a lower correlation strength (Fig. 2, Table 2).

**Table 2:** Reproducibility of feature-tracking analysis.

	Intra-observer variability				Inter-observer variability			
	Mean Bias ± SD	ICC	p-value	Correlation R	Mean Bias ± SD	ICC	p-value	Correlation R
Global circumferential strain	0.27 ± 0.84	0.98	<0.001	0.95	0.04 ± 1.80	0.92	<0.001	0.79
Segmental circumferential strain	2.66 ± 11.34	0.78	<0.001	0.65	1.94 ± 12.91	0.66	<0.001	0.60
Global longitudinal strain	−0.05 ± 0.43	0.95	<0.001	0.94	0.04 ± 1.43	0.92	<0.001	0.99
Segmental longitudinal strain	−1.45 ± 10.03	0.88	<0.001	0.68	1.63 ± 13.14	0.77	<0.001	0.62

ICC: intra-class correlation co-efficient. ‘A sample size of 10 participants was taken per variable.

### Myocardial Strain

Feature-tracking circumferential and longitudinal strain was analyzable for all participants.

#### The influence of sex on myocardial contractility

**Figure 3:**
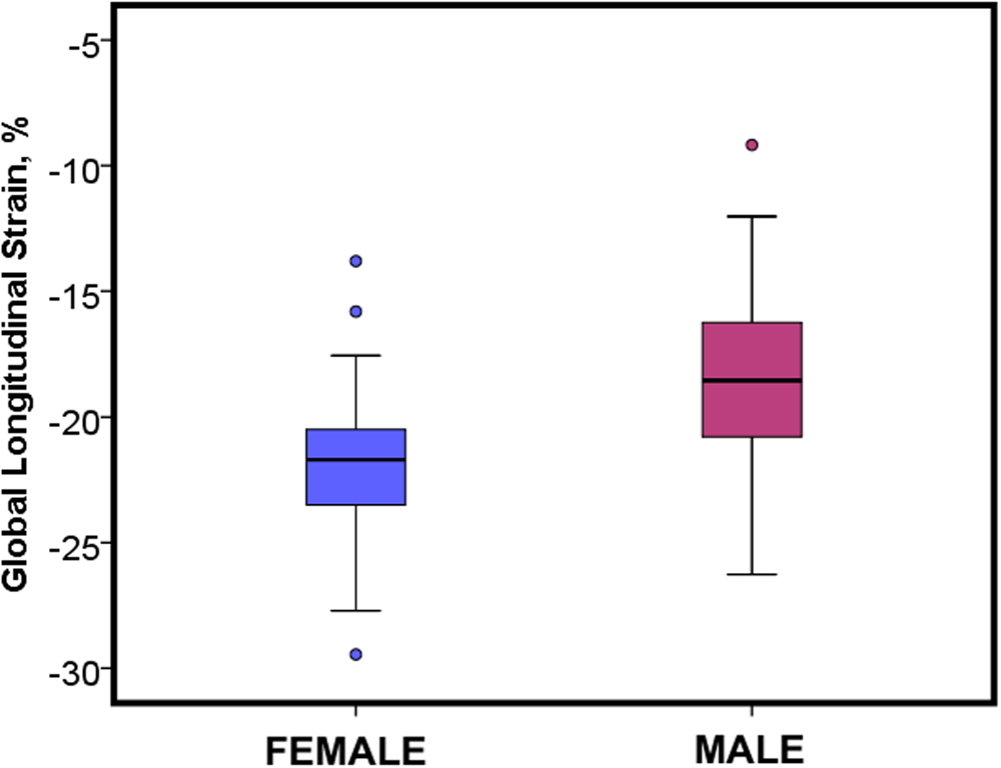
Sex differences in global longitudinal strain assessed by cardiac magnetic resonance feature-tracking analysis.

Female patients had higher magnitudes of global longitudinal strain (Female: −21.91 ± 3.01%, Male: −18.48 ± 3.65%, p = < 0.001, Table 3, Fig. 3). There was a similar trend, with higher magnitudes of strain for females, for all long axis views (LV outflow tract, p < 0.001, Vertical long axis, p = 0.002, Horizontal long axis, p = 0.088).

**Table 3:** Feature-tracking derived longitudinal strain.

	Male (n = 43)		Female (n = 45)		p value
	Mean value ± Standard deviation				
Global Strain	−18.48	±3.65	−21.91	±3.01	<0.001
Horizontal Long Axis	−18.58	±5.06	−20.48	±5.26	0.088
Segment 3	−19.64	±8.95	−17.15	±9.74	0.215
Segment 9	−14.67	±10.23	−12.10	±8.50	0.204
Segment 14	−19.95	±9.98	−21.74	±12.72	0.464
Segment 12	−13.63	±8.82	−16.09	±10.26	0.232
Segment 10	−13.99	±13.62	−20.78	±13.90	0.023
Segment 6	−25.96	±13.22	−30.12	±13.85	0.155
LVOT	−18.32	±6.12	−21.62	±4.92	<0.001
Segment 5	−21.34	±15.26	−33.95	±13.33	<0.001
Segment 11	−12.04	±10.29	−15.45	±10.52	0.131
Segment 16	−20.09	±12.10	−18.46	±12.43	0.538
Segment 14	−18.78	±11.81	−20.54	±12.05	0.493
Segment 08	−17.96	±11.42	−26.11	±13.43	0.003
Segment 02	−24.49	±11.04	−24.39	±13.12	0.969
Vertical Long Axis	−18.55	±4.62	−21.90	±5.12	0.002
Segment 4	−18.88	±8.69	−22.65	±10.70	0.074
Segment 10	−9.54	±10.12	−11.33	±12.45	0.463
Segment 15	−24.64	±11.13	−26.42	±13.81	0.508
Segment 13	−25.84	±15.51	−26.09	±15.19	0.938
Segment 7	−18.16	±13.29	−24.70	±14.99	0.033
Segment 1	−15.02	±11.48	−18.35	±10.49	0.161

Values are presented as mean ± standard deviation. Segments are based on the American heart association 16-segment model. LVOT- left ventricular outflow tract view.

**Figure 4:**
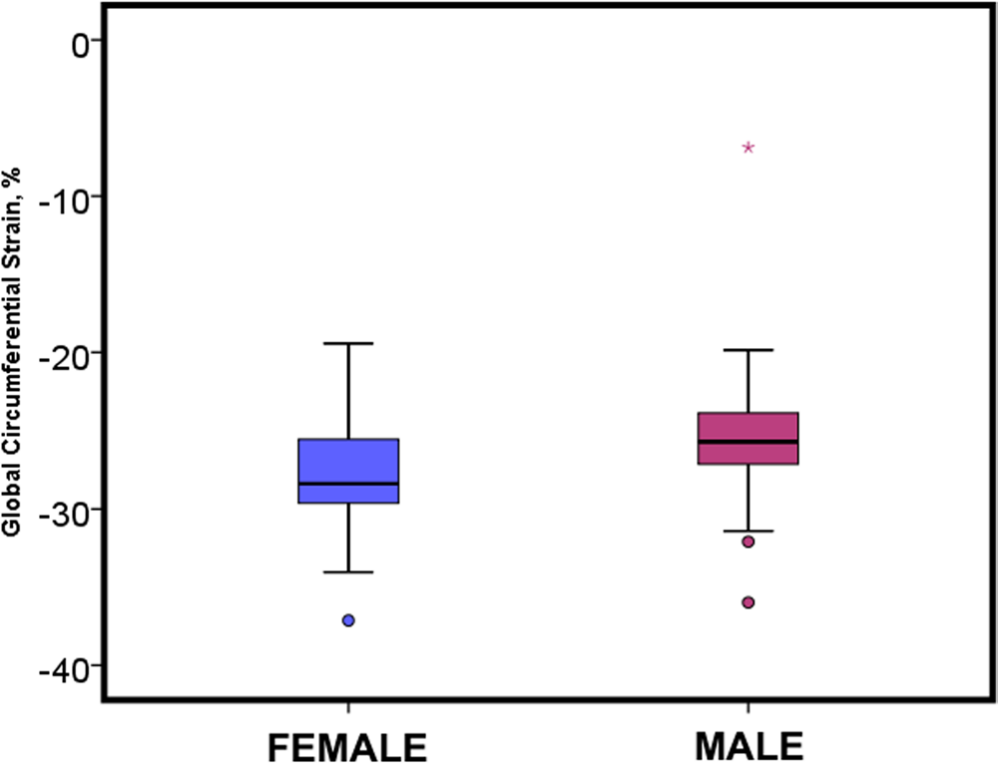
Sex differences in global circumferential strain assessed by cardiac magnetic resonance feature-tracking analysis.

There was no significant difference in global circumferential strain between participants (Female: −27.9 ± 3.48%, Male: −25.41 ± 4.50%, p = 0.643, Table 4, Fig. 4).

**Table 4:** Feature-tracking derived circumferential strain.

	Male (n = 43)		Female (n = 45)		p value
	Mean value ± Standard deviation				
Global Circumferential strain	−25.41	±4.50	−27.94	±3.48	0.643
Basal slice	−27.75	±5.13	−31.93	±3.97	0.495
Segment 1	−23.21	±9.93	−30.31	±10.43	0.491
Segment 2	−25.24	±14.92	−24.84	±10.51	0.165
Segment 3	−25.44	±8.56	−23.84	±10.15	0.404
Segment 4	−20.75	±11.83	−26.79	±10.29	0.831
Segment 5	−27.35	±10.80	−32.66	±7.60	0.225
Segment 6	−31.34	±10.77	−33.06	±9.92	0.534
Mid-LV slice	−27.05	±5.77	−28.77	3.90	0.104
Segment 7	−26.66	±10.18	−29.52	±7.95	0.773
Segment 8	−22.88	±8.27	−25.84	±10.17	0.139
Segment 9	−23.26	±9.42	−25.94	±9.14	0.180
Segment 10	−23.74	±7.32	−26.13	±9.20	0.183
Segment 11	−23.50	±7.51	−26.53	±7.90	0.069
Segment 12	−22.22	±7.56	−23.37	±9.11	0.522
Apical slice	−31.25	±6.94	−35.00	±6.50	0.011
Segment 13	−28.19	±12.08	−28.12	12.35	0.994
Segment 14	−26.37	±11.31	−30.62	±12.26	0.097
Segment 15	−28.68	±11.31	−33.59	±10.96	0.058
Segment 16	−27.75	±11.25	−28.62	±13.77	0.748

Values are presented as mean ± standard deviation. Segments are based on the American heart association 16-segment model.

#### Strain and healthy ageing

Both global circumferential (R = −0.12), and longitudinal strain (R = −0.17) had a poor negative correlation with healthy ageing We carried out linear regression analyses accounting for the effect of sex as well as age. There was no association between global circumferential or longitudinal strain and ageing, after accounting for gender (Table 5).

**Table 5:** Associations of strain, sex and age.

	B	95% CI	p value
	Global circumferential strain		
Age	−0.02	−0.06 to 0.03	0.540
Sex	2.52	0.82 to 4.23	0.004
	**Global longitudinal strain**		
Age	−0.04	−0.75 to 0.01	0.084
Sex	3.42	2.01 to 4.82	<0.001

CI- confidence intervals.

## Discussion

We have investigated the feature-tracking derived circumferential and longitudinal strain in a large sample of healthy volunteers at 3.0 T utilizing commercially available feature-tracking software. The main findings of this study are that:

1. Global circumferential and longitudinal strain have good reproducibility, unlike segmental strain.
2. There is a gender specific difference in magnitudes of longitudinal but not circumferential strain.
3. There are no changes in magnitudes of strain with healthy ageing.

A number of studies have assessed reproducibility of feature-tracking derived strain, in healthy volunteers^20,30^ and in patients^31^. There is concern that unlike global strain^31^, segmental strain derived with feature-tracking is not yet ready for clinical use because of poor reproducibility^2,13,27^. In our study, we identified that inter-observer analyses of segmental strain yielded moderate (ICC = 0.66, segmental circumferential strain) and good (ICC = 0.77 segmental longitudinal strain) reproducibility as assessed by intra-class correlation co-efficient, associated with a significant mean bias and standard deviations, in keeping with what has been previously reported^2,13,27^. This has important implications when it comes to choosing the strain modality to be used in studies investigating regional myocardial biomechanics.

Comparing our values with the literature, we report higher circumferential strain values when compared with healthy volunteer values at 1.5 T (−17 to −25%)^15,26,28,32^, and at 3.0 T (−21.9% to −22.6%)^23^ whilst longitudinal strain values are broadly similar (−19 to −21%)^15,23,26,28,32^. In keeping with previous studies carried out on small numbers of participants (n < 35)^13,20,33^ we report segmental strain has significant variability, with high standard deviations.

In our study we identified that females generate larger magnitudes of longitudinal strain, whilst the observed difference in circumferential strain between different genders was not statistically significant. This result is in keeping with other studies using feature-tracking to look at healthy volunteers. Augustine *et al*.^15^ (n = 145, 37% male), and Taylor *et al*.^26^ (n = 100, 50% male) reported that circumferential strain was not associated with sex, whilst André *et al*.^32^ (n = 150, 50% male) and Liu *et al*.^23^ (n = 130, 46% male), identified a statistically significant difference in circumferential strain between the sexes, with females having larger magnitudes of strain There was a significant difference in longitudinal strain magnitudes between sexes in our study in keeping with Liu *et al*.^23^, Augustine *et al*.^15^, Taylor *et al*.^26^ and André *et al*.^32^. This difference. The difference in strain magnitudes could be partly attributed to the gender specific difference in myocardial volumes^34^ which would imply that greater myocardial shortening would be required to generate similar cardiac output between sexes.

In our study, we did not observe a relationship between feature-tracking strain and ageing. Looking to the literature, André^32^ using feature-tracking, Oxenham^35^ using tagging, and Neizel^36^ using strain-encoded CMR did not identify any association between age and strain. Taylor^26^ reported an age related increase in circumferential but not longitudinal strain, but no information was provided on the potential associations with sex. Kuznetsova *et al*.^37^ using echocardiography in 236 healthy volunteers reported an inverse association between longitudinal strain and age.

In conclusion, we have described circumferential and longitudinal strain at 3.0 Tesla in a reasonably large sample of healthy adults across a broad age range with feature-tracking software. We have observed that longitudinal and circumferential strains varied in a regional distribution with higher strain values in the anterior and lateral LV territories. Longitudinal strain values were higher in females than in males. There was no age related difference in strain after accounting for age.

## Methods

### Study Population

The UK Research Ethics Service (ethics reference 11/AL/0190) approved the study, all of the participants provided written informed consent and all studies were performed in accordance with relevant guidelines^38^. Healthy volunteers aged at least 18 years with no prior medical history (including cardiovascular health problems, medication or systemic illness) were invited to participate by placing advertisements in public buildings (e.g. hospital, university). The other exclusion criteria included standard contraindications to MR (e.g. metallic implants and metallic foreign body) and known or suspected pregnancy. Written informed consent was subsequently obtained from prospective participants. A 12-lead electrocardiogram (ECG) was obtained in all subjects and a normal ECG was an inclusion criterion. Patient characteristics were recorded.

### MR Acquisition

Participants underwent an MRI scans at 3.0 T (MAGNETOM Verio, Siemens Healthcare, Erlangen, Germany) in a university research center. Images were acquired using an anterior phased-array body coil (16-element) and a posterior phased-array spine coil (24-element).

### MR protocol

LV dimensions were assessed using b-SSFP cinematographic breath-hold sequences. Typical imaging parameters are as shown in Table 6. The heart was imaged in multiple parallel short-axis planes 7-mm thick separated by 3 mm gaps, as well as in the 2-chamber, 3-chamber, and 4-chamber long-axis views.

**Table 6:** Typical imaging parameters, at 3.0 T MR field strength.

b-SSFP	3.0 Tesla
TR (ms)	40.6
TE (ms)	1.5
FoV (mm)	340
Flip Angle (degree)	50
Slice Thickness (mm)	7
Resolution (mm)	256 × 256
Bandwidth (Hz/pixel)	977
segments per cardiac frame	16
Shimming method	manual

TR: repetition time (ms); TE: echo time (ms); FoV: field of view (mm).

Participants over 45 years of age had their renal function checked and if the estimated glomerular filtration rate (eGFR) was > 30 mls/min/1.73 m^2^ gadolinium contrast was administered (0.15 mmol/kg per bolus of gadolinium diethyltriaminepenta -acetic acid (Gd-DTPA, Magnevist, Bayer Healthcare). Late gadolinium enhancement images covering the entire LV were acquired 10–15 minutes after intravenous contrast agent administration using segmented phase-sensitive inversion recovery (PSIR) turbo fast low-angle shot sequence.

### Image Analysis

Data sets were anonymised to ensure operators were blinded to all other data. The absence of late gadolinium enhancement (myocardial fibrosis or scar) was determined qualitatively by visual assessment by D.C. (>3 years CMR experience) and C.B. (>10 years CMR experience). The absence of myocardial late gadolinium enhancement was another requirement for inclusion of the data in this analysis.

LV mass and function were analyzed in randomly ordered, de-identified scans by CMR-trained cardiologists using computer-assisted planimetry (Syngo MR®, Siemens Healthcare, Erlangen, Germany) as previously described^27^.

### Feature-tracking analysis

3 long-axis (horizontal long axis, vertical long axis, and left ventricular outflow tract views) and 3 short-axis (basal, mid-LV, apical) slices were chosen per each volunteer. The mid-left ventricular short axis slice was chosen as the equidistant slice between the mitral valve plane and the LV apex.

The LV was segmented using the anterior right ventricular-LV insertion point as the reference point. Diogenes CMR feature-tracking software (TomTec Imaging Systems, Germany) was used to quantify strain from short axis cine images at mid-left ventricular level. The operators derived strain following a standard protocol taught by the software manufacturer^15,39^.

To minimise observer bias, 10 datasets were identified at random and coded using a different code sequence to the main dataset, which was disclosed after the analysis was performed, a week apart by 2 analysts.

### Statistical Analysis

Statistical analysis was performed using SPSS software (SPSS Inc, Chicago, IL, USA, version 22), R V.2.15 or higher (R Foundation for Statistical Computing, Vienna, Austria). Normality was tested using the Shapiro-Wilk test. Continuous variables were expressed as mean ± standard deviation (SD). Student’s t-test was used to compare means. Linear regression was used to investigate the association of age and sex with strain. Inter- and intra- observer reproducibility was assessed using Bland-Altman statistics, intra-class correlation co-efficient and Pearson correlation.

A p-value of <0.05 was considered statistically significant.

### Disclosures

The University of Glasgow holds a research agreement with Siemens Healthcare.
